# The Mucosal Adjuvant Cyclic di-AMP Exerts Immune Stimulatory Effects on Dendritic Cells and Macrophages

**DOI:** 10.1371/journal.pone.0095728

**Published:** 2014-04-22

**Authors:** Ivana Škrnjug, Christine Rueckert, Rimma Libanova, Stefan Lienenklaus, Siegfried Weiss, Carlos A. Guzmán

**Affiliations:** 1 Department of Vaccinology and Applied Microbiology, Helmholtz Centre for Infection Research, Braunschweig, Germany; 2 Department of Molecular Immunology, Helmholtz Centre for Infection Research, Braunschweig, Germany; Commissariat a l'Energie Atomique(cea), France

## Abstract

The cyclic di-nucleotide bis-(3′,5′)-cyclic dimeric adenosine monophosphate (c-di-AMP) is a candidate mucosal adjuvant with proven efficacy in preclinical models. It was shown to promote specific humoral and cellular immune responses following mucosal administration. To date, there is only fragmentary knowledge on the cellular and molecular mode of action of c-di-AMP. Here, we report on the identification of dendritic cells and macrophages as target cells of c-di-AMP. We show that c-di-AMP induces the cell surface up-regulation of T cell co-stimulatory molecules as well as the production of interferon-β. Those responses were characterized by *in vitro* experiments with murine and human immune cells and *in vivo* studies in mice. Analyses of dendritic cell subsets revealed conventional dendritic cells as principal responders to stimulation by c-di-AMP. We discuss the impact of the reported antigen presenting cell activation on the previously observed adjuvant effects of c-di-AMP in mouse immunization studies.

## Introduction

To better manage health risks and costs, modern vaccines are no longer made from whole pathogens. Rather, they contain antigenic subunits that mainly provide the immune target to elicit memory responses against a broad spectrum of the pathogen's strains and clades. For efficacy, most subunit vaccines require additional factors, so-called adjuvants; among them are pathogen-associated molecular patterns (PAMPs). Vaccine adjuvants can compensate for the lack of danger signals in subunit-based formulations, thereby improving the activation of innate and adaptive immune responses. Cyclic di-nucleotides are one such group of promising candidate adjuvants [1]. They are signaling molecules which are involved in critical processes such as attachment and biofilm formation in prokaryotes and they control cell motility and proliferation states in the protozoon dictyostelium [2]–[5]. Recently, an additional cyclic di-nucleotide, cyclic [G(2′,5′)pA(3′,5′)p] (cGAMP), was reported to activate the stimulator of interferon genes (STING) and to be synthesized by the mammalian enzyme cyclic GMP-AMP synthase (cGAS) upon stimulation with foreign DNA [6]–[9]. Cyclic di-nucleotides such as bis-(3′,5′)-cyclic dimeric adenosine monophosphate (c-di-AMP), bis-(3′,5′)-cyclic dimeric guanosine monophosphate (c-di-GMP), and bis-(3′,5′)-cyclic dimeric inosine monophosphate (c-di-IMP) proved to have immune modulatory activity in mice and humans [10]–[17]. We and others have previously shown that c-di-AMP, c-di-GMP, and c-di-IMP act as potent adjuvants in immunization experiments with mice [12], [16], [18], [19]. We demonstrated that c-di-AMP promotes humoral as well as cellular immune responses to model and vaccine antigens in mice immunized via the mucosal route. Immune modulation by c-di-AMP was observed to contribute to a balanced T_H_1/T_H_2/T_H_17 response [18]. Splenocytes from immunized mice re-stimulated *in vitro* with antigen in the presence of c-di-AMP showed enhanced proliferation activity. Furthermore, we demonstrated that *in vitro* antigen presentation by murine bone marrow-derived dendritic cells (BMDCs) results in increased T cell proliferation in the presence of c-di-AMP [18]. This last observation suggests a stimulatory effect of c-di-AMP on dendritic cells (DCs) leading to T cell activation. Additional studies demonstrated the capacity of cyclic di-nucleotides to induce the expression of type I interferons (IFNs) via activation of the axis: stimulator of IFN genes/TANK-binding kinase 1/IFN response factor 3 (STING/TBK1/IRF3) [13], [15], [20]–[22]. However, it is still unknown to which extent type I IFNs are responsible for adjuvanticity, as well as in which cell subtype(s) their production is specifically induced.

Pathogen-evoked immune stimulation and subsequent antigen processing and presentation is usually accompanied by PAMP signaling leading to activation and surface expression of T cell co-stimulatory molecules on antigen presenting cells (APCs). Only then do APCs become effective in priming T cells, an important step toward adaptive immunity and, eventually, memory. There is limited knowledge on the effector functions of c-di-AMP on immune cells, such as DCs. By and large, previous studies have been restricted to the murine system, they have addressed neither all APCs nor specific cell subsets, and they were generally limited to *in vitro* models. Here we asked which APCs are targeted by the putative PAMP mimicking effects of c-di-AMP that caused the observed T cell expansion [18]. To this end, we analyzed the effects of c-di-AMP application on different immune cells. We characterized the surface expression of co-stimulatory molecules and the production of type I IFN as hallmarks of innate immune activation and signaling leading to adaptive immune responses. The observed c-di-AMP effects were also confirmed for human immune cells. Our studies show that DCs and macrophages (MΦs) respond to c-di-AMP by exhibiting enhanced surface expression of T cell co-stimulatory molecules and IFN-β production. We further demonstrate the preferential activation of conventional DCs, known as principal stimulators of antigen-specific T cell responses.

## Results

### The c-di-AMP effect on T cell proliferation is not direct

**Figure 1:**
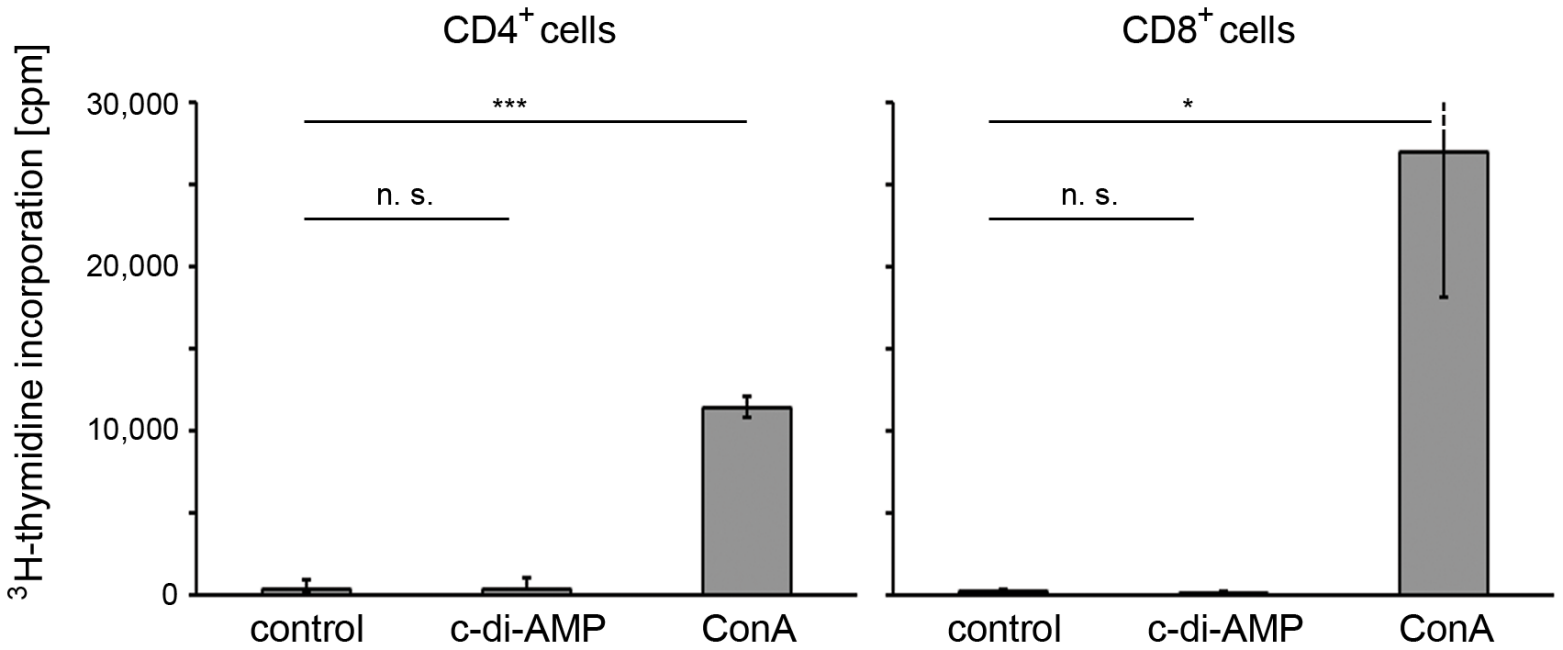
C-di-AMP does not directly affect murine T cell proliferation *in vitro*. Murine splenocytes were sorted for CD4^+^ and CD8^+^ T cells (CD62L^high^, CD44^low^, CD25^−^) and treated with either 5 µg/ml c-di-AMP, 5 µg/ml concanavalin A (ConA; positive control), or left untreated (control) in the presence of ^3^H-thymidine. As a measure for proliferation, ^3^H-thymidine incorporation in T cells was determined by scintillation. The error bars show SEM for n = 3. Differences were statistically significant at *p*<0.001 (***), *p*<0.05 (*) or non-significant (n. s.) with respect to the control.

First, we asked if the c-di-AMP-induced T cell proliferation that we reported previously [18] is an effect of the direct action of c-di-AMP on T cells, for example as a super antigen, in the absence of potential mediator cells. Murine CD4^+^ and CD8^+^ T cells were isolated from splenocyte preparations and incubated for 4 days with c-di-AMP. Significantly enhanced T cell proliferation, as assessed by ^3^H-thymidine incorporation, was found after stimulation with concanavalin A (positive control) but not after c-di-AMP treatment (Figure 1). Based on this finding, further experiments focused on the identification of cells mediating the effect of c-di-AMP on T cell proliferation.

### C-di-AMP affects surface expression of T cell receptor co-stimulatory molecules on murine DCs and MΦs *in vitro*

**Figure 2:**
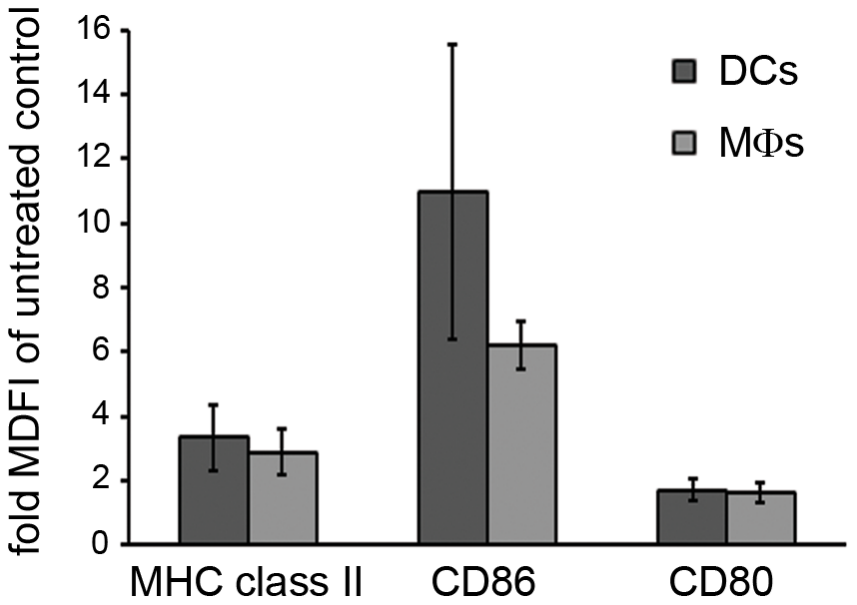
C-di-AMP activates murine immune cells *in vitro*. Murine bone marrow derived model dendritic cells (DCs, CD11c^+^) and model macrophages (MΦs, CD11b^+^) were incubated for 24 h with 5 µg/ml c-di-AMP or medium without additives (control). Cells were decorated with fluorescently labeled antibodies against MHC class II and the co-stimulatory molecules CD80 and CD86. The diagram shows the normalized median fluorescence intensity (fold increase as compared to the control) analyzed by flow cytometry. Error bars are SEM for n = 3.

Activated APCs are characterized by up-regulated surface expression of major histocompatibility complex (MHC) class II and the co-stimulatory molecules CD80 and CD86. To test if c-di-AMP directly up-regulates these molecules, murine BMDCs and bone marrow-derived MΦs were exposed for 24 h to c-di-AMP *in vitro*. Cell surface CD80, CD86, and MHC class II (I-A) were detected with fluorescently labeled antibodies and analyzed by flow cytometry. C-di-AMP treatment induced an up-regulation of surface MHC class II, CD80, and CD86 on DCs and MΦs when compared to cells grown in medium without additive (Figures 2 and S1).

### C-di-AMP application activates preferentially DCs of the murine nose-associated lymphoid tissue (NALT) and the mediastinal lymph nodes (MLN) *in vivo*

**Figure 3:**
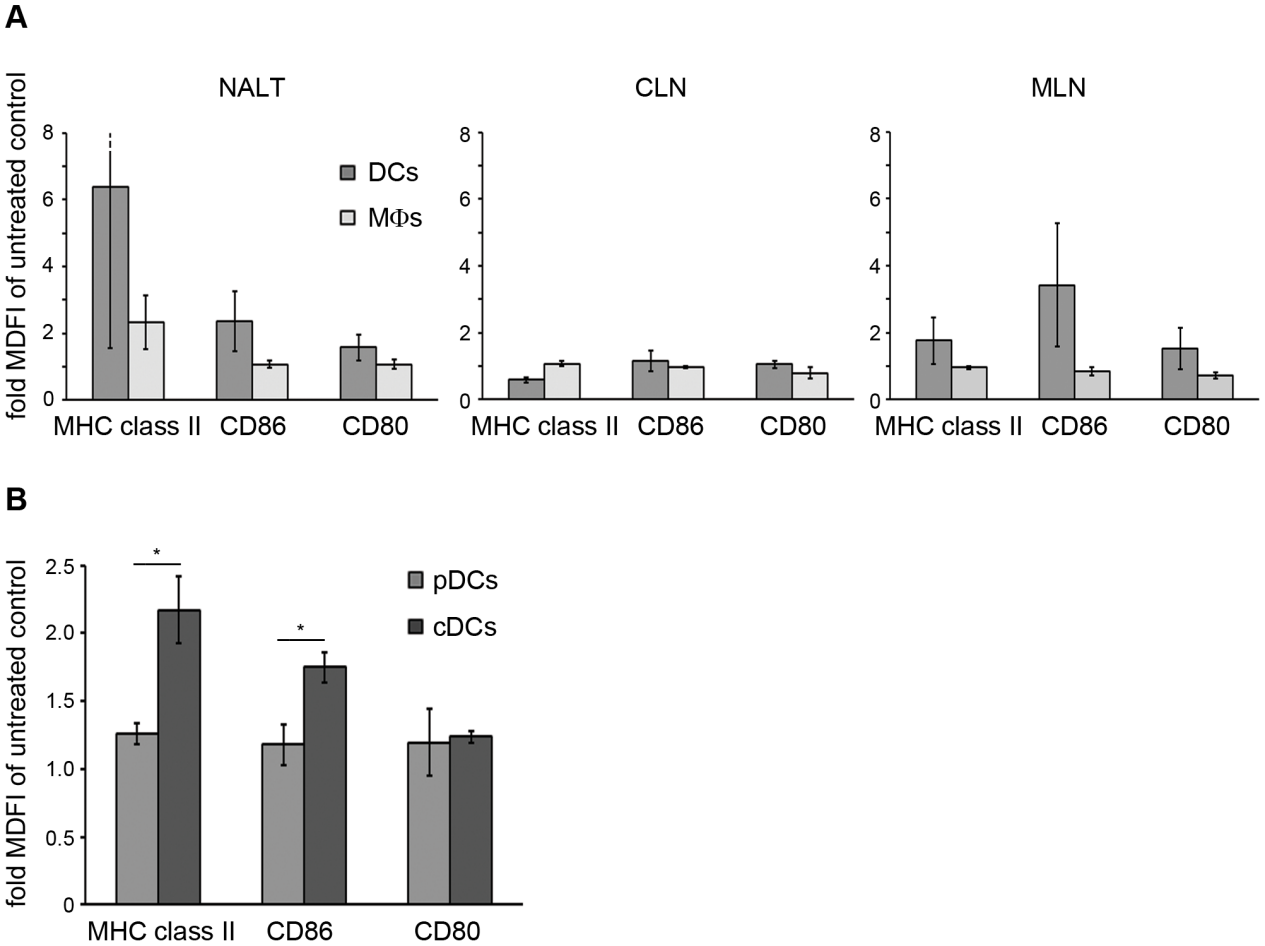
C-di-AMP up-regulates T cell co-stimulatory molecules preferentially on conventional murine dendritic cells (DCs). (**A**) *In vivo* effects of c-di-AMP on mouse APCs. Flow cytometric analysis of DCs (CD11c^+^) and MΦs (CD11c^−^, CD11b^+^) from the nose-associated lymphoid tissue (NALT), the cervical lymph nodes (CLN) or the mediastinal lymph nodes (MLN) 24 h after i. n. administration of c-di-AMP in mice. (**B**) *In vitro* effects of c-di-AMP on mouse DCs. Murine bone marrow derived model conventional (cDCs) and plasmacytoid DCs (pDCs) were generated by culturing in the presence of Flt3l and incubated for 24 h with 5 µg/ml c-di-AMP or without additives (control). Cells were decorated with fluorescently labeled antibodies against identification markers CD11c (DCs), CD11b (cDCs), B220 (pDCs), and MHC class II, CD80 and CD86. The diagrams show the normalized median fluorescence intensity (fold increase as compared to the control) analyzed by flow cytometry. Error bars are SEM for n = 3. Differences are statistically significant at *p*<0.01 (**) or *p*<0.05 (*).

Our experiments demonstrated that c-di-AMP can directly activate DCs and MΦs *in vitro*. However, the experimental conditions would not necessarily match the architectural complexity of an inductive site or in terms of pharmacokinetics the *in situ* active concentration of c-di-AMP. Thus, we investigated if c-di-AMP is able to activate those APCs *in vivo* under the same conditions in which it exerts its adjuvant properties. To this end, c-di-AMP was intra-nasally (i. n.) administered to mice. After 24 h, DCs and MΦs from the NALT, the MLN and the cervical lymph nodes (CLN) were prepared and the surface expression of CD80, CD86 and MHC class II was analyzed by flow cytometry. Upon i. n. administration of c-di-AMP, the expression of MHC class II, CD80 and CD86 was clearly up-regulated in DCs of the NALT and the MLN (Figures 3A and S2A).

### Murine conventional DCs are the principal c-di-AMP responder DC subset *in vitro*

Since it is known that DCs occur in functionally distinct subsets, we tested the response of murine DC subsets to c-di-AMP application *in vitro*. BMDCs were cultured in the presence of FMS (Feline McDonough Sarcoma Virus) like tyrosine kinase 3 ligand (Flt3l) to give rise to plasmacytoid and conventional DC subsets. Treatment with c-di-AMP for 24 h led to up-regulation of MHC class II and the co-stimulatory molecules CD80 and CD86 preferentially in conventional DCs (Figures 3B and S2B).

### C-di-AMP application in mice leads to IFN-β reporter gene activation in DCs and the MΦ/monocyte/granulocyte population, but not in T and B cells

**Figure 4:**
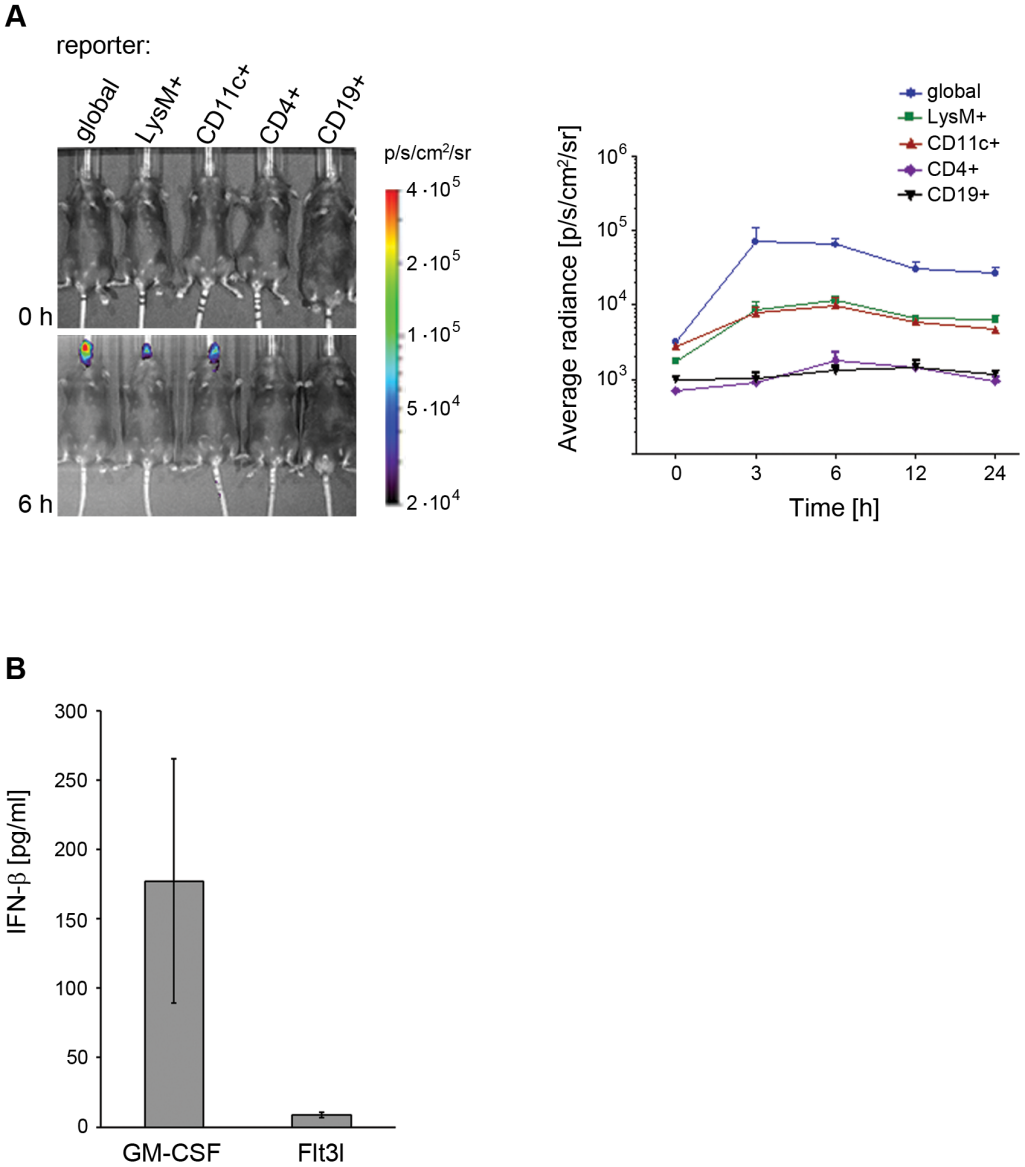
C-di-AMP induces IFN-β production in murine dendritic cells (DCs). (**A**) C-di-AMP targets the *IFNB* promoter in DCs and monocytes/macrophages/granulocytes *in vivo*. Mice of indicated phenotypes were i. n. treated with c-di-AMP: “CD4+” indicates T cell specific, “CD19+” B cell specifc, “LysM+” monocyte/macrophage/granulocyte specific, and “CD11c+” DC specific control of luciferase expression by the IFN-β promoter. Quantification of *in vivo* imaging signals derived from luciferase activity at different time points is shown for n = 5. Results are expressed as average of radiance (in photons/s/cm^2^/steridian). Error bars are SEM. (**B**) C-di-AMP induces IFN-β production in DCs *in vitro*. BMDCs were cultured in the presence of GM-CSF or Flt3l, as indicated on the x axis. IFN-β secretion was determined in the culture medium by ELISA. Error bars are SEM, n = 3.

Activation of different immune cell types was further investigated *in vivo* by employing conditional IFN-β reporter mice. These transgenic mice provide the option to position a luciferase reporter under the control of the *IFNB* promoter by crossing them with mice expressing Cre recombinase under tissue or cell specific promoters. In this study we used mice with an activated reporter function in CD19^+^ B cells, CD4^+^ T cells, CD11c^+^ DCs, and LysM^+^ MΦs/monocytes/granulocytes. The tissue/cell specific luciferase expression was measured *in vivo* at different time points after i. n. administration of c-di-AMP in mice. Luciferase activity was detected as early as 3 h post administration of c-di-AMP, peaked after 6 h and was strongly reduced after 24 h in both DCs and the MΦ/monocyte/granulocyte population (Figure 4A). The kinetics observed in the cell subset specific reporter mice closely matched the one of the global reporter animal. Furthermore, studies performed using IFN receptor knockout mice carrying the reporter gene showed that reporter activation was independent of a paracrine activation loop (data not shown). Interestingly, the detected IFN-β reporter responses were strictly localized in the nasal tissue region. Since IFN-β production is a known hallmark of immune cell activation, these results strongly support DCs and MΦs as the main early responders to c-di-AMP *in vivo*. There are no reporter mice which allow us to dissect IFN-β activation in different DC subsets. Thus, *in vitro* studies were performed to further analyze the c-di-AMP induced IFN-β response of different DC subsets. To this end, bone marrow derived monocytes were cultured i) in the presence of granulocyte macrophage colony-stimulating factor (GM-CSF) resulting in an exclusively conventional DC population or ii) in the presence of Flt3l resulting in a mixed population of conventional and plasmacytoid DCs. After treatment with c-di-AMP, IFN-β was readily detected in the medium of DCs cultured in the presence of GM-CSF, whereas lower concentrations were observed in supernatant fluids of DCs cultured in the presence of Flt3l (Figure 4B). Hence, conventional DCs seem to be the major IFN-β producers upon stimulation with c-di-AMP.

### C-di-AMP induces up-regulation of T cell co-stimulatory molecules and IFN-β production in human DCs

**Figure 5:**
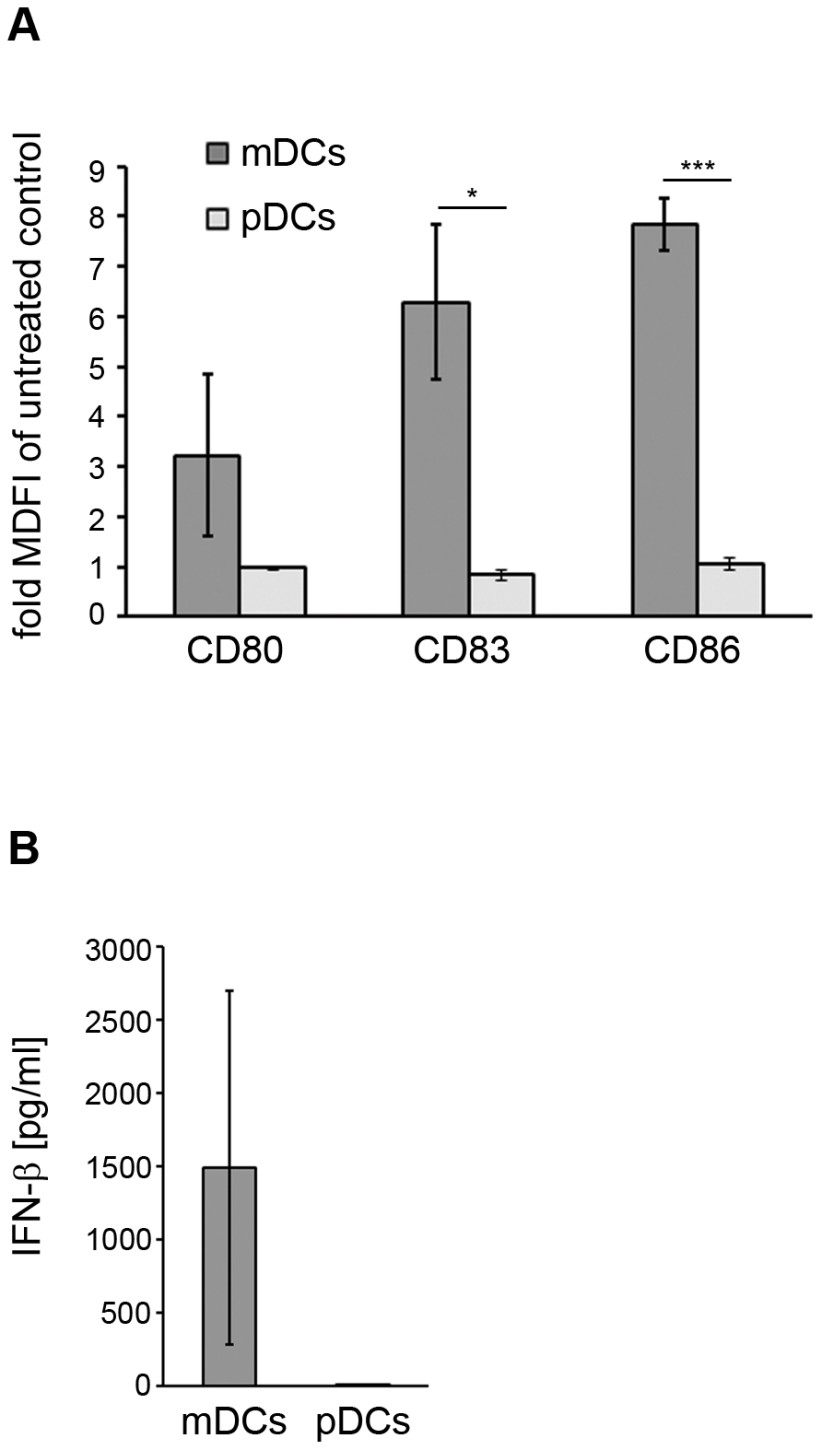
C-di-AMP preferentially activates human myeloid dendritic cells (DCs) *in vitro*. PBMC-derived human plasmacytoid DCs (pDC) or myeloid (conventional) DCs (mDC) were incubated for 24 h in the presence of 60 µg/ml c-di-AMP or without additive (control). (**A**) Cells were decorated with fluorochrome-conjugated antibodies specific for the identification markers CD11c (mDC), CD303 (pDC) CD80, CD83 and CD86, and analyzed by flow cytometry. Normalized median fluorescence intensity (MDFI) is shown as fold increase compared to the control. Error bars show SEM for n = 3. (**B**) The medium from the cultured mDC or pDC was analyzed for IFN-β secretion by ELISA. Error bars show SEM for n = 3. Differences are statistically significant at *p*<0.001 (***) or *p*<0.05 (*).

We next investigated if human DCs exhibit a similar response to c-di-AMP. To this end cultured human peripheral blood mononuclear cell (PBMC)-derived myeloid and plasmacytoid DCs were treated for 24 h with c-di-AMP or left untreated (control). Then, the expression of surface receptors critical for cross talk with T cells was analyzed by flow cytometry, and IFN-β secretion was evaluated by ELISA. Flow cytometry revealed that CD80, CD83 and CD86 expression was preferentially up-regulated in myeloid DCs but was virtually absent on plasmacytoid DCs after stimulation by c-di-AMP (Figures 5A and S3). Similarly to what was observed for murine cells, myeloid DCs were the major contributors to IFN-β secretion following c-di-AMP treatment (Figure 5B). Taken together, the myeloid DC subset (representing the conventional DC subset in human PBMCs) is the preferential responder to c-di-AMP (Figure 5).

## Discussion

Adjuvants can modulate different steps involved in the elicitation of an adaptive immune response to an antigen. However, there is only fragmentary knowledge on the underlying molecular mechanisms of action of most adjuvants. The discovery of the role of PRRs (pattern recognition receptors) in PAMP binding and signaling revealed more details about the functional mode of action of PAMP-like adjuvant molecules, preferentially in cells of the innate immune system. Numerous PRRs and their ligands are described and their importance for adjuvant efficacy was investigated [23], [24]. For example, various PRR signaling pathways induce the expression of type I IFNs, which are important immune modulators [25], [26]. However, the current state of knowledge on mechanisms of adjuvant activity is far from explaining their observed immunological effector functions. Such information would accelerate the development of vaccines that promote immune responses able to efficiently clear specific pathogens [27]. A better understanding of the molecular basis of adjuvanticity could also promote the rational design of successful vaccines by supplementing them with the appropriate adjuvants or combinations thereof [27].

Here, we studied the effector functions of the candidate adjuvant c-di-AMP on different immune cells *in vitro* as well as *in vivo*. We asked which cell types undergo activation and which cellular processes and molecules are involved. We previously reported that c-di-AMP promotes antigen specific T cell proliferation [18]. C-di-AMP does not seem to directly exert this effect on T cells (Figure 1), thus we concluded that the observed T cell activation is mediated by other immune cell types. This was also suggested by previous *in vitro* studies showing that DCs which were antigen-loaded in the presence of c-di-AMP were more efficient at promoting the activation of antigen-specific T cells from TCR transgenic mice [18]. Accordingly, we examined the response of different types of APCs to c-di-AMP. We showed that treatment with c-di-AMP induces the up-regulation of the expression of MHC class II as well as T cell co-stimulatory molecules on the surface of both murine and human DCs and MΦs *in vitro* (Figures 2, 3B and 5A). These *in vitro* findings were further confirmed by *ex vivo* studies in which murine DCs and MΦs were analyzed after i. n. c-di-AMP application (Figure 3A). 24 h after i. n. administration especially DCs and not so much MΦs were activated by c-di-AMP in the lymphoid compartments. Although early DC antigen presenting activity in the draining CLN was described for i. n. immunization experiments with viral antigens [28], our adjuvant candidate c-di-AMP alone did not notably induce T cell stimulatory molecules on APCs of the CLN after 24 h. The more pronounced DC activation was observed in the NALT, the lymphoid tissue directly associated with the site of administration, and also in the MLN. The early DC response in the MLN is in line with several studies reporting a similar observation upon i. n. immunization with model antigens and adjuvants [29]–[31] as well as upon i. n. infection with influenza virus [32]. We also demonstrated that murine DCs and MΦs/monocytes/granulocytes respond to *in vivo* administration of c-di-AMP by the up-regulation of IFN-β expression (Figure 4A).

Taken together, we observed the effects of c-di-AMP on its target cells on two different aspects of immune regulation. First, we found up-regulation of CD80 and CD86 on the surface of mouse and human DC subsets treated with c-di-AMP (Figures 3B and 5A). These molecules are known to interact with CD28 on the T cell surface to deliver co-stimulatory signals after TCR engagement with the antigen presenting MHC molecule. This co-stimulation represents an important regulator of T cell immune tolerance toward activation in response to pathogenic invasion, for example by promoting T cell proliferation and differentiation. Nevertheless, both CD80 and CD86 are also ligands for the T cell surface molecule CTLA-4 which facilitates negative T cell activation signaling. As opposed to the constitutively surface-expressed CD28, CTLA-4 is only transported to the surface upon TCR-mediated activation. It is believed to have a role in the prevention of an overshooting T cell proliferation to control antigen-specific T cell-mediated immune responses. In an effort to find the major c-di-AMP target DC subset we identified conventional DCs as the principal responders (Figures 3B and 5A). This points to an adjuvant mechanism in which c-di-AMP facilitates T cell activation by CD80 and CD86-mediated co-stimulation through the classical APC type that mainly functions in naïve T cell activation. In conjunction with an antigen in a subunit vaccine, c-di-AMP would provide the means to overcome cellular immune tolerance as a first step to an adaptive response that eventually leads to the establishment of specific memory cells.

Second, we showed that IFN-β production, a downstream indicator of PRR signaling pathway activation, was induced *in vivo* in DCs as well as in the MΦ/monocyte/granulocyte population but not in B or T cells (Figure 4). It has been reported that murine MΦs respond to c-di-AMP secreted by *Listeria monocytogenes* with the production of IFN-β [17], [33]. Our data generated with a reporter mouse system confirm MΦs as candidate c-di-AMP responders and extend these findings to DCs as major contributors to the IFN-β response to c-di-AMP *in vivo*. Our results would also fit in line with the Tip-DC IFN-β response reported for murine listeriosis, if the reported IFN-β gene induction were mediated by *Listeria*-released c-di-AMP [34], [35]. The observed IFN-β reporter response was locally restricted to nasal tissue regions (Figure 4A). This largely excludes CD11c-positive MΦs as contributors to the response of the CD11c specific reporter signal, because such MΦs are lung (alveolae)-associated [36]. This is in contrast to what was observed with other immune stimulators (*e.g*. poly I:C) for which the luciferase signal also occurred in the liver or the spleen [37]. The locally restricted effect of i. n. applied c-di-AMP could be of advantage in order to reduce the potential risk for toxic side effects at the systemic level.

Our results suggest that c-di-AMP acts on the level of PRR signaling pathways in innate immune cells. This is supported by reports describing c-di-AMP and c-di-GMP as activators of IFN-β production via a pathway that involves the adaptor/sensor STING, TBK-1 and IRF3 [13], [15], [20]–[22]. In addition it was reported that c-di-GMP induces the production of TNF-α *via* a STING-dependent but IFN type I independent pathway [38]. Several immune effects of IFN-β are described [25], [39]. The up-regulation of cytokines, chemokines and intermediate signaling molecules can modulate immune cell activity. The specific effect of IFN-β correlates with cell state, timing, amount and the molecular context of its encounter. IFN-β can differentially modulate signal transducer and activator of transcription (STAT) signaling in monocytes, T cells, and B cells to affect their transcriptional, differentiation, proliferative, apoptotic, and pro-inflammatory activity. It is known that type I IFNs can regulate effector T cell function and differentiation of T_H_1, T_H_2, T_H_17 and T_reg_ cells [25], [26], [39]. Hence, it is conceivable that the IFN-β-inducing effect of c-di-AMP contributes, at least in part, to the reported proliferation of antigen-specific T cell types and the evolvement of the proposed balanced T_H_1/T_H_2/T_H_17 cell response [18]. DC subset targeting was investigated also with regard to the c-di-AMP-induced IFN-β production. Conventional DCs showed a much more pronounced IFN-β production than plasmacytoid DCs (Figures 4B and 5B). This finding was somewhat surprising because plasmacytoid DCs are known to be specialized in IFN type I production and usually produce these IFNs in much higher amounts than conventional DCs, which are specialized in antigen presentation. By targeting conventional DCs, c-di-AMP evokes the secretion of a rather limited amount of IFN-β which may be important to fine tune immune responses. Interestingly, also the IFN-β response to *L. monocytogenes* was reported not to be mediated by plasmacytoid DCs either [34], [35]. Since it is known that the bacterium secretes c-di-AMP in the course of infection [17], [33], our results further strengthen the suggestion that c-di-AMP is indeed the mediator of the *L. monocytogenes*-induced IFN-β.

The modes of action described here do not exclude additional, not yet identified mechanisms of c-di-AMP-mediated immune response modulation. For example, other co-stimulatory molecules or secreted immune signaling molecules could be regulated in a c-di-AMP-dependent manner to enhance or modulate antigen-induced immune responses by acting on either effector or bystander cells. However, our results further elucidate intermediate steps of the immune response cascade leading to the immune modulatory activity of c-di-AMP observed in immunization studies on mice [18]. They advance the knowledge on modes of adjuvant action toward the regulation of effective immunization responses.

## Materials and Methods

### Mice/Ethics statement

Female BALB/c (H-2d) or C57BL/6 (H-2b) mice 6–8 weeks old were purchased from Harlan (Rossdorf, Germany). The generation of the global and tissue specific IFN-β reporter mice has been previously described [34], [40]. They were bred at the animal facilities of the Helmholtz Centre for Infection Research under specific pathogen-free conditions. All animal experiments in this study have been performed in agreement with the local government of Lower Saxony, Germany (No. 33.11.42502-04-017/08). Animals were randomly assigned to experimental groups.

### Synthesis of c-di-AMP

C-di AMP was prepared as previously described [18]. Briefly, c-di-AMP was synthesized by cyclization and purified by Reversed Phase HPLC. The chemical structure was confirmed by ^1^H- and ^13^P-nuclear magnetic resonance and matrix-assisted laser desorption/ionization mass spectrometry. For the use in experiments, lyophilized c-di-AMP was dissolved in water. Lipopolysaccharide (LPS) contamination was ruled out by employing the HEK-Blue LPS Detection Kit (InvivoGen, San Diego, California, USA).

### Preparation of murine bone marrow-derived cells

The femur and tibia from two 6-8 week old C57BL/6 or BALB/c mice (Harlan, Rossdorf, Germany) per experiment were flushed with medium (RPMI 1640 supplemented with 10% fetal calf serum, 100 U/ml penicillin, 50 µg/ml streptomycin, 100 µg/ml gentamycin; Gibco, Grand Island, New York, USA) to collect bone marrow cells. Erythrocytes were lysed in 150 mM NH_4_Cl, 10 mM KHCO_3_, 0.1 mM ethylenediaminetetraacetic acid (EDTA) (Sigma-Aldrich, Steinheim, Germany), pH 7.2. The remaining bone marrow cells were seeded at 10^6^ cells/ml in medium supplemented with either 5 ng/ml murine GM-CSF (BD Pharmingen, Franklin Lakes, New Jersey, USA) and cultured for 7 days or with 100 ng/ml murine Flt3l (BD Pharmingen, Franklin Lakes, New Jersey, USA) and cultured for 9 days or with 10 ng/ml murine M-CSF (eBioscience Inc., San Diego, California, USA) and cultured for 7 days at 37°C with 5% CO_2_ in a humidified atmosphere. The bone marrow-derived cells cultured in the presence of GM-CSF give rise to non-adherent *in vitro* conventional DC model populations, positive for the marker CD11c. Flt3l promotes the differentiation into a mixed population of *in vitro* model conventional DCs and plasmacytoid DCs, both positive for CD11c with the plasmacytoid DCs being additionally positive for B220, while the *in vitro* model MΦs cultured in the presence of M-CSF are positive for CD11b [41]-[43].

### Preparation of cells from the murine NALT, CLN and MLN

To isolate APCs from the NALT, cells were scratched from the nasal cavity of five animals per group. The combined cells were transferred to medium (RPMI 1640 supplemented with 10% fetal calf serum, 100 U/ml penicillin, 50 µg/ml streptomycin, 100 µg/ml gentamycin; Gibco, Grand Island, New York, USA), pressed through a 100 µm pore nylon mesh, and erythrocytes were lysed in 150 mM NH_4_Cl, 10 mM KHCO_3_, 0.1 mM ethylenediaminetetraacetic acid (EDTA) (Sigma-Aldrich, Steinheim, Germany), pH 7.2. To isolate APCs from the CLN or the MLN, lymph nodes were also transferred to medium (see above), pressed through a 100 µm pore nylon mesh and washed.

### *In vitro* stimulation of cells

The culture medium of cells was supplemented with 1, 5 or 60 µg/ml c-di-AMP or 0.1 or 1 µg/ml LPS (Sigma-Aldrich, Steinheim, Germany) or 10 µg/ml CpG (InvivoGen, San Diego, California, USA) or left without additive. Cells were incubated for 24 h at 37°C and 5% CO_2_ in a humidified atmosphere and were then analyzed.

### T cell proliferation assay

Spleens were isolated from mice, transferred to medium (RPMI 1640 supplemented with 10% fetal calf serum, 100 U/ml penicillin, 50 µg/ml streptomycin, 100 µg/ml gentamycin; Gibco, Grand Island, New York, USA), and gently pressed through a 100 µm cell mesh. Erythrocytes were lysed in 150 mM NH_4_Cl, 10 mM KHCO_3_, 0.1 mM EDTA (pH 7.2) and cells were washed. T cells were decorated with fluorescently labeled antibodies against CD44, CD62L, CD25, CD4, and CD8 (see below) and sorted for CD44^low^, CD62L^high^, CD25^−^, CD4^+^ or CD44^low^, CD62L^high^, CD25^−^, CD8^+^ cell populations, respectively. Cells were incubated at 37°C and with 5% CO_2_ for 4 days in the presence of either 5 µg/ml c-di-AMP, 5 µg/ml concanavalin A (Sigma-Aldrich, Steinheim, Germany) or left untreated. Cells were then incubated for another 16 h in the presence of 10 µCi/ml ^3^H-thymidine. Incorporated ^3^H-thymidine was measured by a γ scintillation counter (1450 Microbeta Trilux, Wallach Sverige, Upplands Vasby, Sweden).

### *In vivo* stimulation in mice (i. n. c-di-AMP application)

Female 6–8 week old C57BL/6 or BALB/c mice (Harlan, Rossdorf, Germany) were anesthetized with Isoflurane (Abbott Animal Health, Abbott Park, Illinois, USA) and treated i. n. (10 µl per nostril) with 5 µg per dose of c-di-AMP in Ampuwa (Serumwerk, Bernburg, Germany) or with Ampuwa alone in the control group. 24 h after the i. n. application mice were sacrificed and tissue/cells were collected from five animals per group.

### Preparation of human DCs from PBMCs

Human DCs were prepared from the blood of healthy human donors who provided informed consent. The blood donors' health is rigorously checked before being admitted for blood donation. This process includes a national standardized questionnaire with health questions, an interview with a medical doctor and standardized laboratory tests for HIV1/2, HBV, HCV and syphilis infections and hematological cell counts. The blood donations were obtained from the Institute for Clinical Transfusion Medicine, Klinikum Braunschweig, Germany, in accordance with the rules of the regional Ethics Committee of Lower Saxony, Germany, and the declaration of Helsinki and were analyzed anonymously. PBMCs were isolated by centrifugation over a Ficoll (GE Healthcare, Uppsala, Sweden) cushion and myeloid and plasmacytoid DCs were prepared by negative selection using the Myeloid DC Isolation Kit, human, and the Plasmacytoid DC Isolation Kit II, human, (Miltenyi, Bergisch Gladbach, Germany), respectively. Isolated cells were recovered overnight in medium (RPMI 1640 supplemented with 10% fetal calf serum, 100 U/ml penicillin, 100 µg/ml streptomycin; Gibco, Grand Island, New York, USA) at 37°C and with 5% CO_2_ in a humidified atmosphere.

### Flow cytometric analysis of immune cell markers

After stimulation cells were pre-incubated with Fc receptor blocking anti-mouse CD16/CD32 (clone 93) or human Fc receptor binding inhibitor (eBioscience Inc., San Diego, California, USA), and decorated with select fluorophore-conjugated antibodies out of the following: CD80 (clone 16-10A1, APC-conjugated), CD11b (clone M1/70, eFluor450-conjugated), CD8 (clone 53-6.7, APC-conjugated), CD44 (clone IM7, Pacific Blue-conjugated), CD4 (clone RM4-5, PE-Cy7-conjugated) (eBioscience Inc., San Diego, California, USA) or anti-mouse CD86 (clone GL1, Brilliant Violet 605-conjugated), CD11c (clone N418, PE-Cy7-conjugated), anti-human CD80 (clone 2D10, Brilliant Violet 650-conjugated), CD83 (clone HB15e, PE-conjugated), CD86 (clone IT2.2, Brilliant Violet 605-conjugated) (BioLegend, San Diego, California, USA) or anti-mouse CD86 (clone GL1, PE-conjugated), I-A^b^ (clone AF6-120.1, FITC-conjugated), B220 (clone RA3-6B2, PE-conjugated), CD62L (clone MEL-14, FITC-conjugated), CD25 (clone PC61, PE-conjugated) (BD Pharmingen, Franklin Lakes, New Jersey, USA). Human plasmacytoid DCs were identified by the marker CD303 (using PE-Cy7-conjugated anti-human CD303a, clone 201A; eBioscience Inc., San Diego, California, USA), myeloid DCs by the marker CD11c (using Brilliant Violet 711-conjugated anti-human CD11c, clone 3.9; BioLegend, San Diego, California, USA) [44], [45]. A blue fluorescent amine-reactive dye (Invitrogen, Carlsbad, California,USA; L23105) was routinely used as live/dead cell marker in flow cytometric analyses. FACS analysis was performed using an LSR-II and with FACSDiva software (BD Bioscience, Franklin Lakes, New Jersey, USA) and the evaluation software FlowJo Mac v9.6 (Tree Star, Inc., Ashland, Oregon, USA).

### IFN-β reporter mouse assay

To study the effect of c-di-AMP on the induction of IFN-β genes C57BL/6 IFN-ββ-luc reporter mice (see also paragraph “Mice”, above) were used. The conditional reporter mice received c-di-AMP by the i. n. route at a concentration of 5 µg per mouse. IFN-β gene induction was analyzed by measuring luciferase activity as a reporter by *in vivo* imaging. To this end, mice were injected intravenously with 150 mg/kg of D-luciferin firefly (Synchem, Felsberg/Altenburg, Germany) dissolved in PBS at time points 0, 3, 6, 12 and 24 h after administration of c-di-AMP. Mice were anesthetized with Isofluran (Curamed, Karlsruhe, Germany) and monitored using the IVIS 200 imaging system (CaliperLS, Perkin Elmer Inc., Waltham, Massachusetts, USA). Photon flux was quantified with the LivingImage 3.2 Software (CaliperLS, Perkin Elmer Inc., Waltham, Massachusetts, USA) and is expressed in photons/s/cm^2^/steradian.

### IFN-β detection in cell culture medium

Murine IFN-β was analyzed using the Legend Max Mouse IFN-β ELISA kit with pre-coated plates (BioLegend, San Diego, California, USA); human IFN-β was analyzed using the VeriKine human IFN-β ELISA kit (PBL Interferon Source, Piscataway, New Jersey, USA). Detection was performed by light absorbance measurement at a wavelength of 450 nm using a Synergy 2 Multi-Mode Microplate Reader (BioTek, Winooski, Vermont, USA).

### Statistical Analysis

The statistical analysis was performed by applying the unpaired t-test; n.s. indicates not significant; *** indicates p<0.001; ** indicates p<0.01; and * indicates p<0.05.

## Supporting Information

Figure S1 **C-di AMP-stimulated murine DCs and MΦs respond with the up-regulation of CD80, CD86 and MHC class II.** BMDCs or MΦs were incubated for 24 h in the presence of 5 µg/ml c-di-AMP or without additive (untreated control). Cells were decorated with fluorochrome-conjugated antibodies specific for CD80, CD86 and MHC class II, and analyzed by flow cytometry. The TLR ligand LPS was used as control stimulator to assess if the cells were generally activatable. Histograms of flow cytometry measurements represent a single experiment. The data collected from multiple experiments are summarized in figure 2. The x axis displays fluorescence intensity (at a logarithmic scale) that was recorded with fluorescent antibodies against the indicated molecules CD80, CD86 or MHC class II. The signals derive from living singlet cells and are selected by gating based on forward/sideward scatter (for singlet cell identification) and fluorescent live/dead marker (low intensity on live cells) and fluorescent antibody (high intensity) analysis.
(TIF)

Figure S2 **C-di-AMP up-regulates T cell co-stimulatory molecules preferentially on conventional murine dendritic cells (DCs).** (**A**) 24 h after i. n. application of c-di-AMP in mice DCs and macrophages (MΦs) were isolated from nose-associated tissue (NALT), cervical lymph nodes (CLN) or mediastinal lymph nodes (MLN) and decorated with fluorochrome-conjugated antibodies specific for the identification markers of DCs (CD11c^+^) or MΦs (CD11b^+^, CD11c^−^), and for CD80, CD86, MHC class II and analyzed by flow cytometry. (**B**) Bone marrow derived *in vitro* model DCs were grown in the presence of Flt3l and stimulated for 24 h in the presence of 5 µg/ml c-di-AMP. They were decorated with fluorochrome-conjugated antibodies specific for the identification markers CD11c (DCs), CD11b (conventional DCs, cDCs), B220 (plasmacytoid DCs, pDCs), CD80 and CD86, then analyzed by flow cytometry. Histograms of flow cytometry measurements represent a single experiment. The data collected from multiple experiments are summarized in figure 3. The x axis displays fluorescence intensity (on a logarithmic scale) that was recorded with fluorescent antibodies against the indicated molecules CD80, CD86 or MHC class II. The signals are derived from living singlet cells positive for the indicated identification marker and are selected by gating based on forward/sideward scatter (for singlet cell identification) and fluorescent live/dead marker (low intensity on live cells) and fluorescent antibody (high intensity) analysis.
(TIF)

Figure S3 **C-di-AMP stimulated human myeloid (conventional) DCs (mDCs) but not plasmacytoid DCs (pDCs) respond with the up-regulation of surface CD80, CD83 and CD86.** PBMC-derived human pDCs or mDCs were incubated for 24 h in the presence of 60 µg/ml c-di-AMP or without additive (untreated control). Cells were decorated with fluorochrome-conjugated antibodies specific for the identification markers CD11c (mDC), CD303 (pDC) and CD80, CD83, CD86 and analyzed by flow cytometry. The TLR ligands LPS (for mDCs) and CpG (for pDCs) were used as control stimulators to if the cells were generally activatable. Histograms of flow cytometry measurements represent a single experiment with the DC subsets originating from one and the same donor. The data collected from multiple donors are summarized in figure 5a. The x axis displays fluorescence intensity (at a logarithmic scale) that was recorded with fluorescent antibodies against the indicated molecules CD80, CD83 or CD86. The signals derive from living singlet cells positive for the DC subset identification marker and are selected by gating based on forward/sideward scatter (for singlet cell identification) and fluorescent live/dead marker (low intensity on live cells) and fluorescent antibody (high intensity) analysis.
(TIF)
