# Supplementary figures and images for: The Mucosal Adjuvant Cyclic di-AMP Exerts Immune Stimulatory Effects on Dendritic Cells and Macrophages
Source: PLoS One. 2014 Apr 22;9(4):e95728. doi: 10.1371/journal.pone.0095728 (PMC3996008; doi:10.1371/journal.pone.0095728)

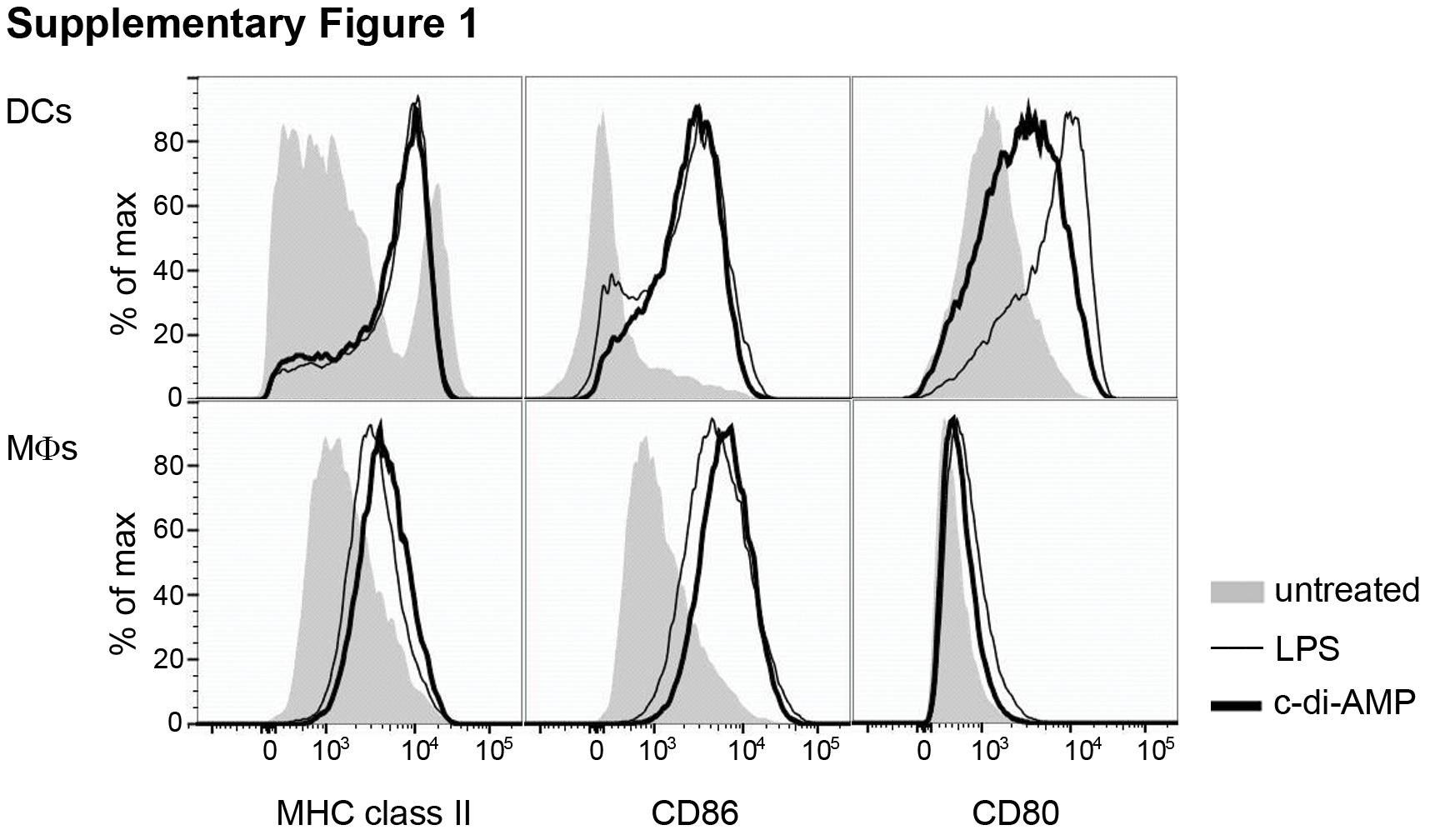

Supplement: Figure S1 — C-di AMP-stimulated murine DCs and MΦs respond with the up-regulation of CD80, CD86 and MHC class II. BMDCs or MΦs were incubated for 24 h in the presence of 5 µg/ml c-di-AMP or without additive (untreated control). Cells were decorated with fluorochrome-conjugated antibodies specific for CD80, CD86 and MHC class II, and analyzed by flow cytometry. The TLR ligand LPS was used as control stimulator to assess if the cells were generally activatable. Histograms of flow cytometry measurements represent a single experiment. The data collected from multiple experiments are summarized in figure 2. The x axis displays fluorescence intensity (at a logarithmic scale) that was recorded with fluorescent antibodies against the indicated molecules CD80, CD86 or MHC class II. The signals derive from living singlet cells and are selected by gating based on forward/sideward scatter (for singlet cell identification) and fluorescent live/dead marker (low intensity on live cells) and fluorescent antibody (high intensity) analysis. (TIF) [file pone.0095728.s001.tif]

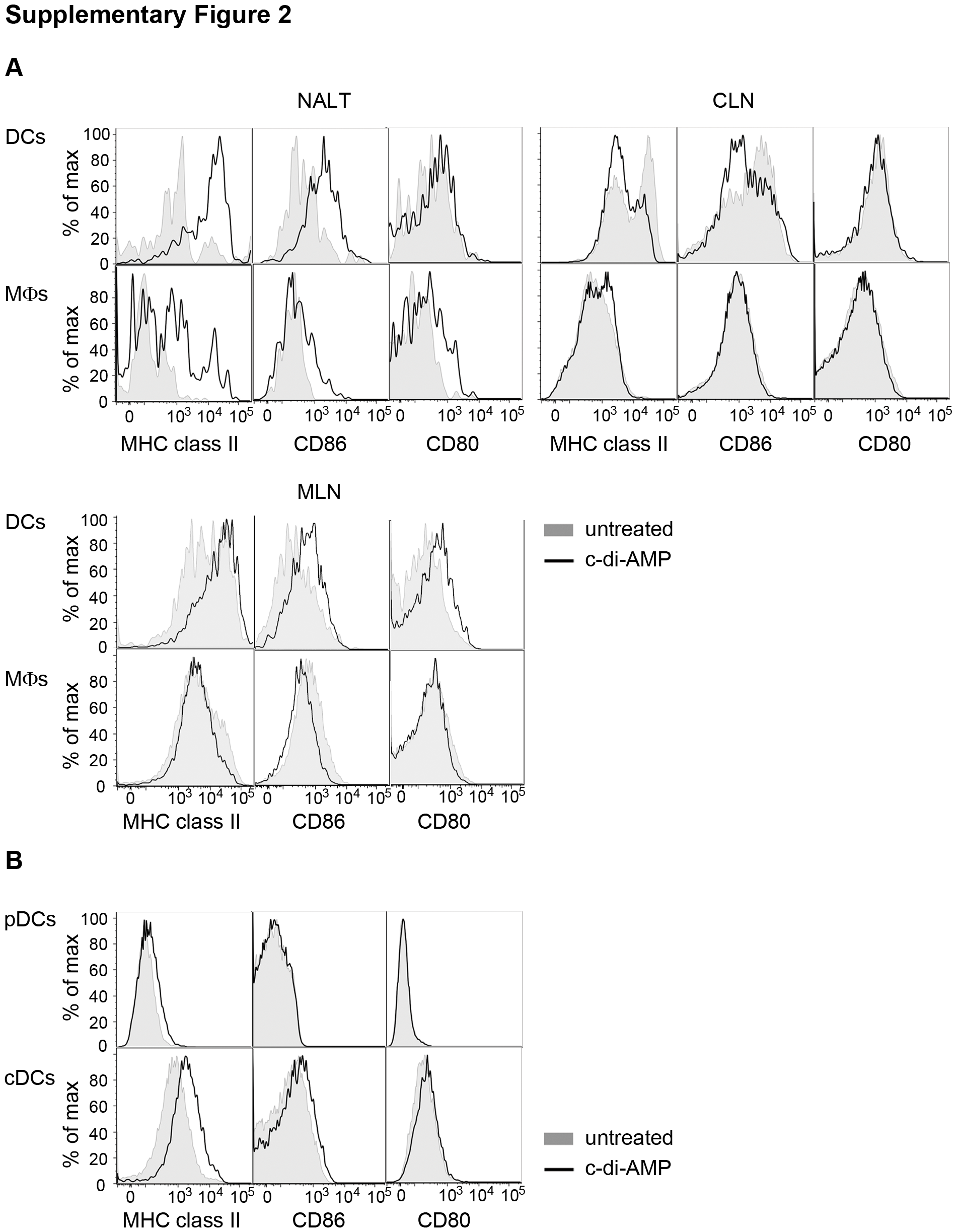

Supplement: Figure S2 — C-di-AMP up-regulates T cell co-stimulatory molecules preferentially on conventional murine dendritic cells (DCs). (A) 24 h after i. n. application of c-di-AMP in mice DCs and macrophages (MΦs) were isolated from nose-associated tissue (NALT), cervical lymph nodes (CLN) or mediastinal lymph nodes (MLN) and decorated with fluorochrome-conjugated antibodies specific for the identification markers of DCs (CD11c+) or MΦs (CD11b+, CD11c−), and for CD80, CD86, MHC class II and analyzed by flow cytometry. (B) Bone marrow derived in vitro model DCs were grown in the presence of Flt3l and stimulated for 24 h in the presence of 5 µg/ml c-di-AMP. They were decorated with fluorochrome-conjugated antibodies specific for the identification markers CD11c (DCs), CD11b (conventional DCs, cDCs), B220 (plasmacytoid DCs, pDCs), CD80 and CD86, then analyzed by flow cytometry. Histograms of flow cytometry measurements represent a single experiment. The data collected from multiple experiments are summarized in figure 3. The x axis displays fluorescence intensity (on a logarithmic scale) that was recorded with fluorescent antibodies against the indicated molecules CD80, CD86 or MHC class II. The signals are derived from living singlet cells positive for the indicated identification marker and are selected by gating based on forward/sideward scatter (for singlet cell identification) and fluorescent live/dead marker (low intensity on live cells) and fluorescent antibody (high intensity) analysis. (TIF) [file pone.0095728.s002.tif]

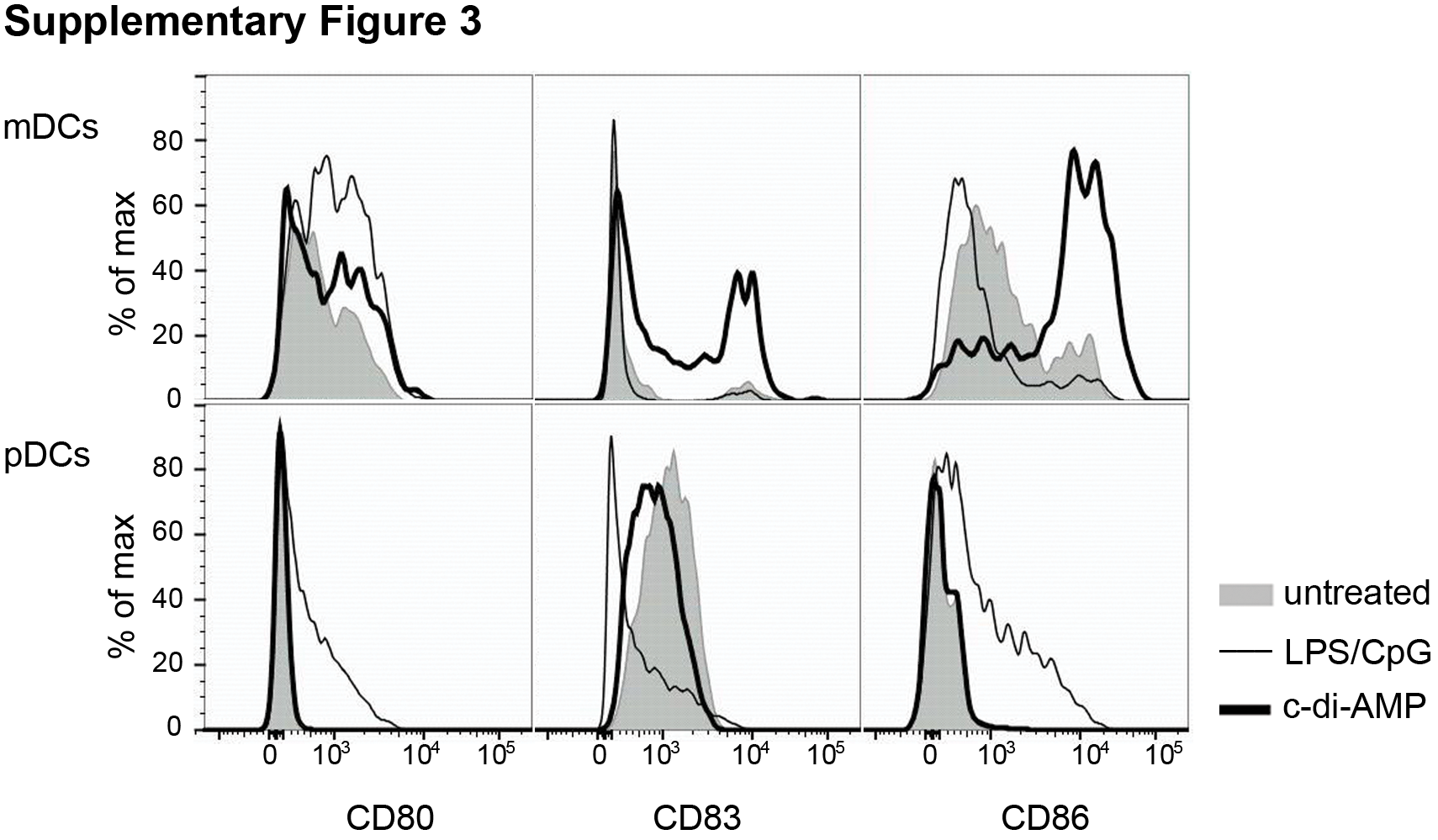

Supplement: Figure S3 — C-di-AMP stimulated human myeloid (conventional) DCs (mDCs) but not plasmacytoid DCs (pDCs) respond with the up-regulation of surface CD80, CD83 and CD86. PBMC-derived human pDCs or mDCs were incubated for 24 h in the presence of 60 µg/ml c-di-AMP or without additive (untreated control). Cells were decorated with fluorochrome-conjugated antibodies specific for the identification markers CD11c (mDC), CD303 (pDC) and CD80, CD83, CD86 and analyzed by flow cytometry. The TLR ligands LPS (for mDCs) and CpG (for pDCs) were used as control stimulators to if the cells were generally activatable. Histograms of flow cytometry measurements represent a single experiment with the DC subsets originating from one and the same donor. The data collected from multiple donors are summarized in figure 5a. The x axis displays fluorescence intensity (at a logarithmic scale) that was recorded with fluorescent antibodies against the indicated molecules CD80, CD83 or CD86. The signals derive from living singlet cells positive for the DC subset identification marker and are selected by gating based on forward/sideward scatter (for singlet cell identification) and fluorescent live/dead marker (low intensity on live cells) and fluorescent antibody (high intensity) analysis. (TIF) [file pone.0095728.s003.tif]
